# Molecular detection of some zoonotic tick-borne pathogens in ticks collected from camels (*Camelus dromedarius*) as hosts and wild rodents as potential reservoirs

**DOI:** 10.1007/s11259-024-10488-9

**Published:** 2024-08-15

**Authors:** Ayat Yousery, Doaa E. Soliman, A. A. Samy, Ahmad M. Allam, Mona G. Shaalan, Amira E. Abdel Hamid

**Affiliations:** 1 Entomology Department, Faculty of Science, Ain Shams University, Cairo, Egypt; 2 Department of Microbiology and Immunology, Veterinary Research Institute, National Research Centre, Dokki, Egypt; 3 Department of Parasitology and Animal Diseases, Veterinary Research Institute, National Research Centre, Dokki, Egypt

**Keywords:** Camels, Ticks, Rodents, Tick-borne pathogens, Egypt

## Abstract

Ticks and tick-borne pathogens pose a great threat to human and animal health. The present study aimed to determine the prevalence of ticks that infest camels and investigate the presence of tick-borne pathogens in the blood of camels, associated ticks, and surrounding rodents as reservoirs. From 100 inspected camels, from different localities in the Giza governorate, 1000 ixodid ticks were collected; these ticks belonged to three genera: *Hyalomma*, *Amblyomma*, and *Rhipicephalus*. The genus *Hyalomma* was represented by four species, *Hyalomma dromedarii* was the most prevalent species (55.4%), followed by *Hyalomma excavatum* (22%), *Hyalomma impeltatum* (11.6%) and *Hyalomma rufipes* (2.8%). The genus *Amblyomma* was represented by two species, *Amblyomma gemma* (2.8%) and *Amblyomma marmoreum* (2.7%), while the genus *Rhipicephalus* was represented by only one species, *Rhipicephalus pulchellus* (2.7%). Ticks, camel blood, and rodents (total number 100 brown rats) are screened for tick-borne pathogens (*Borrelia burgdorferi*, *Borrelia miyamotoi*, *Babesia sp*., and *Coxiella burnetii*) using PCR. Camel blood was found to be infected with *Borrelia burgdorferi* (66.6%), *Borrelia miyamotoi* (55%), and *Babesia* sp. (11.6%). *Coxiella burnetii* DNA was detected in all the collected ticks but was not detected in the blood of camels or rodents. *Borrelia miyamotoi* was detected in 12.5% of *H. impeltatum*, 55% of Camels, and 6% of the rodents, which may indicate a proposed risk of dispersal of *B. miyamotoi*, the agent of tick-borne relapsing fever.

**Supplementary Information:**

The online version contains supplementary material available at 10.1007/s11259-024-10488-9.

## Introduction

Ticks are obligate ectoparasites that rely on blood meals for sustenance. They infest a wide range of hosts, including both wild and domestic animals, as well as humans. Within the phylum Arthropoda, ticks are the most important vectors of disease, second only to mosquitoes (Hoogstraal 1985; de la Fuente et al. 2008). Ticks and tick-borne pathogens (TBPs) can pose serious threats to people, farm animals, pets, and wildlife worldwide (Yin and Luo 2007). Ticks directly impact a vast range of animals, such as cattle, through irritating bites, blood loss, skin damage, and anorexia, all of which result in decreased growth and consequently influence stakeholders (Jabbar et al. 2015). Ticks can transmit a variety of parasitic, viral, and bacterial infections, both zoonotic and nonzoonotic (Torina et al. 2020). Numerous zoonotic bacteria of significant public health concern have been previously identified in tick populations infesting camels and in the blood of camels (Sazmand et al. 2019).

Camels are susceptible to infestation by several hematophagous arthropods, which can have a substantial impact on milk and meat production and may contribute to increased morbidity and mortality rates within camel populations. Several species of ixodid and argasid ticks are frequently reported on camels (Wernery et al. 2014); among these, the genus *Hyalomma* stands out as the most prevalent and significant vector (Elghali and Hassan 2009; Nourollahi Fard et al. 2012).

Rodents (order Rodentia) are major zoonotic disease reservoirs (Han et al. 2015; Olival et al. 2017). Rodents, which make up 42% of the world’s mammalian population, are key reservoirs of diseases that cause zoonotic infections and are among the main causes of many major epidemics (Mawanda et al. 2020). Rodent population numbers sometimes shift seasonally or annually, which can lead to a change in human infection risk (Davis and Calvet 2005).

The importance of rodents in the transmission of zoonoses is a result of their ecology. They are found in every biotope on the planet, and they can breed quickly, eat a broad variety of foods, and adapt to rapid environmental changes (Eisen et al. 2014). Rodents are excellent hosts for a variety of ectoparasites, the majority of which are vectors of socioeconomically important infections (Allam et al. 2019). Rodents serve as important vehicles for the transmission of infectious agents from natural to modified environments in diverse ways. Their role as reservoirs is particularly significant, as they act as hosts for intermediate vectors, facilitating the acquisition of pathogens and dissemination of infectious organisms to humans and other animals (Eisen et al. 2014).

Intense commingling of rodents in spots such as abattoirs and live animal markets offers opportunities to observe disease spread at sites where animals are in intimate connection with hematophagous arthropods involved in disease transmission. Infections produced by mixed tick-borne microbiota are prevalent in nature, and research and epidemiological studies reveal that infections induced by mixed tick-borne microbiota can alter pathogenicity and disease burden in a variety of hosts (Awad et al. 2020).

In the Middle East, there is a scarcity of information about camel hemoparasites in tandem with rodents and their impact on health and productivity. The region’s arid climate, the high prevalence of tick vectors and extensive livestock mobility across neighboring countries create an environment vulnerable to tick-borne diseases. Moreover, the absence of a comprehensive, large-scale integrated tick control program further exacerbates this vulnerability. As a result, camel owners experience substantial economic losses, and concerns regarding human health arise due to the potential transmission of zoonotic pathogens.

Accordingly, this study aimed to fill this gap in knowledge, particularly in Egypt, and molecularly characterize the intracellular tick-borne hematogenous pathogens of sub-clinically infected camelids and related ticks as well as mixed rodents around them.

## Methods

### Sampling procedures

#### Ticks

Ticks were collected during spring/summer 2021 from different localities in the Giza governorate; 100 camels were inspected for tick infestations during the study period. Ticks were manually collected from camels at random using blunt forceps, carefully placed in sterile plastic vials with loose caps and subsequently transported to the laboratory in a dry ice box. In the laboratory, the collected ticks were washed twice with sterile normal saline to remove any remaining particulate contamination from the animal’s skin and then rinsed once with 70% ethanol prior to morphological identification. Ticks were examined using a stereoscope microscope (BOECO, Germany) and identified into genera and species by using appropriate identification keys of morphological shapes (Hoogstraal 1956; Walker et al. 2003; Bakheit et al. 2012; Estrada-Peña et al. 2018). After identification, the ticks were carefully transferred to sterile vials and preserved at -20 °C until further processing.

### Blood sampling

#### Camel blood

During the same period of tick collection, one hundred heparinized camel blood samples were collected from the jugular vein. Blood samples were collected in tubes coated with EDTA. Blood samples were transported to the laboratory in an icebox and stored at − 20 °C until use.

#### Rodent blood

A total of 100 brown rats were live trapped from localities surrounding the inspected camels used in the study. Traps were pre-baited and were placed along walls, on rodent runways, near rodent burrows and at other activity sites. The rats were captured at night and then were conveyed to the laboratory the next morning. The animals were euthanized by carbon dioxide gas asphyxiation, and additional deaths were confirmed by cervical dislocation. (AVMA guidelines for the euthanasia of animals: 2020 edition) https://www.avma.org/resources-tools/avma-policies/avma-guidelines-euthanasia-animals. To collect blood from the rats, a representative number of the rats bled after euthanasia and necropsy from the heart directly.

### Molecular detection of tick-borne pathogens

#### DNA isolation from ticks and blood

DNA was extracted from each morphologically identified tick sample after being crushed in a mortar with liquid nitrogen into small pieces. DNA was extracted from ticks using a Quick-DNA™ Miniprep Plus Kit (Cat no. D4068) and from blood using a GeneJet whole-blood genomic 100xn Thermo Scientific according to the manufacturer’s protocol. The DNA concentration from each sample was quantified by using a NanoDrop spectrophotometer, and the DNA was stored at -20 °C. until use in the PCR assay.

#### Polymerase chain reaction

**Fig. 1:**
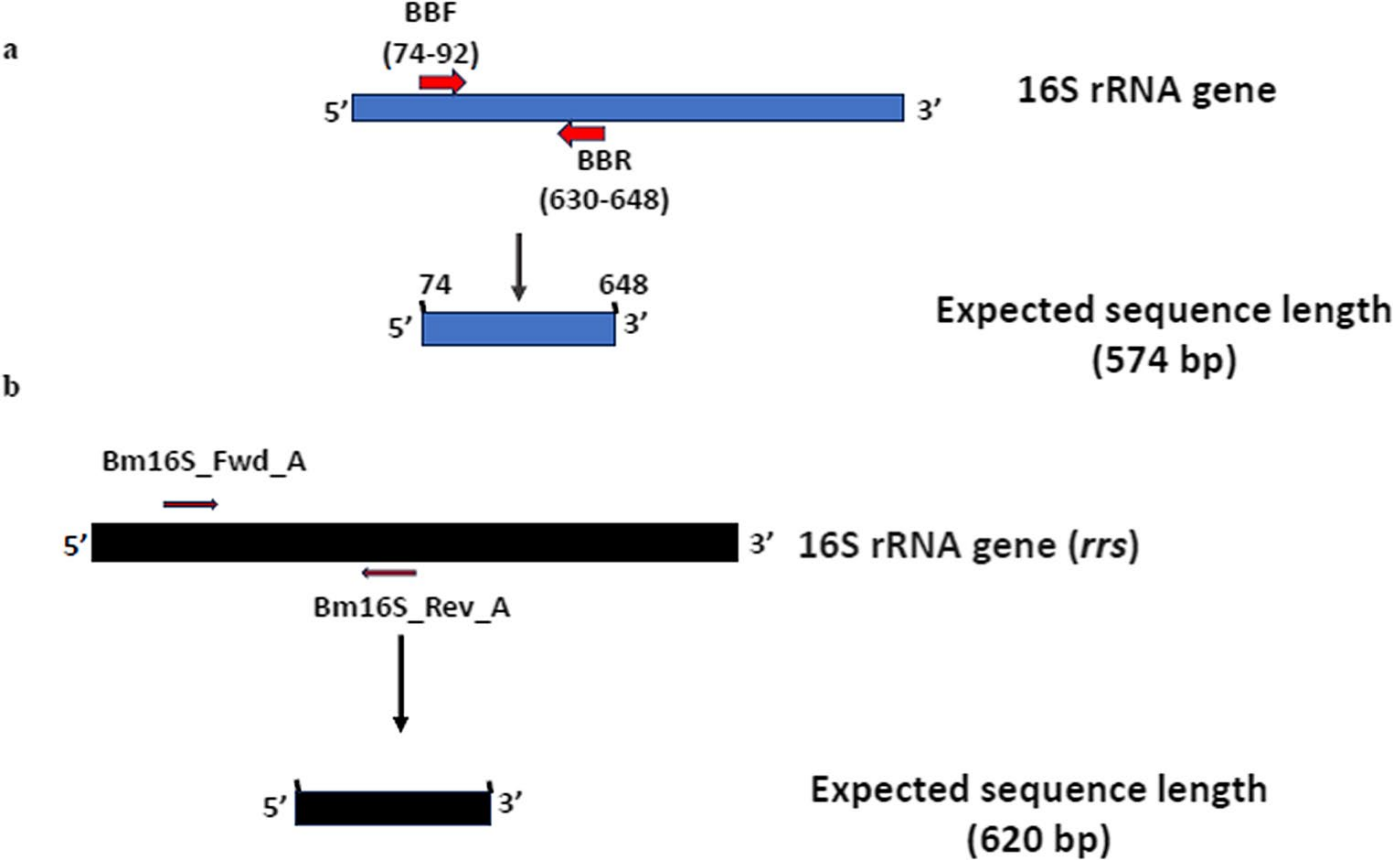
**a** Diagrammatic representation of the expected amplified region of the 16 S rRNA gene in *Borrelia burgdorferi* showing the forward and reverse primer positions (Marconi and Garon 1993). **b** Diagrammatic representation of the expected amplified region of the 16 S rRNA gene (rrs) in Borrelia miyamotoi showing the forward and reverse primer positions (Reiter et al. 2015)

**Fig. 2:**
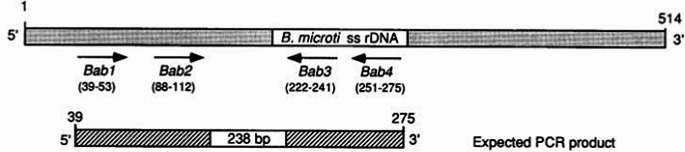
*Babesia sp*. small subunit rDNA sketch showing the expected amplified region with selected forward and reverse primers (Persing et al. 1992)

PCR amplification was performed to detect three pathogens in collected ticks and from the blood of rats and camels using amaR OnePCR (GeneDireX, Inc., USA). Samples were examined for the detection of *B. burgdorferi* using conventional PCR with a primer set of BbF and BbR for the 16 S rRNA gene (Fig. 1A) (Marconi and Garon 1993). The glpQ gene of *Borrelia miyamotoi* was amplified according to Reiter et al. (2015) (Fig. 1B), and *Babesia sp*. were detected by amplifying small-subunit rDNA using oligonucleotide primers (Fig. 2) (Persing et al. 1992; Wei et al. 2001).

**Fig. 3:**
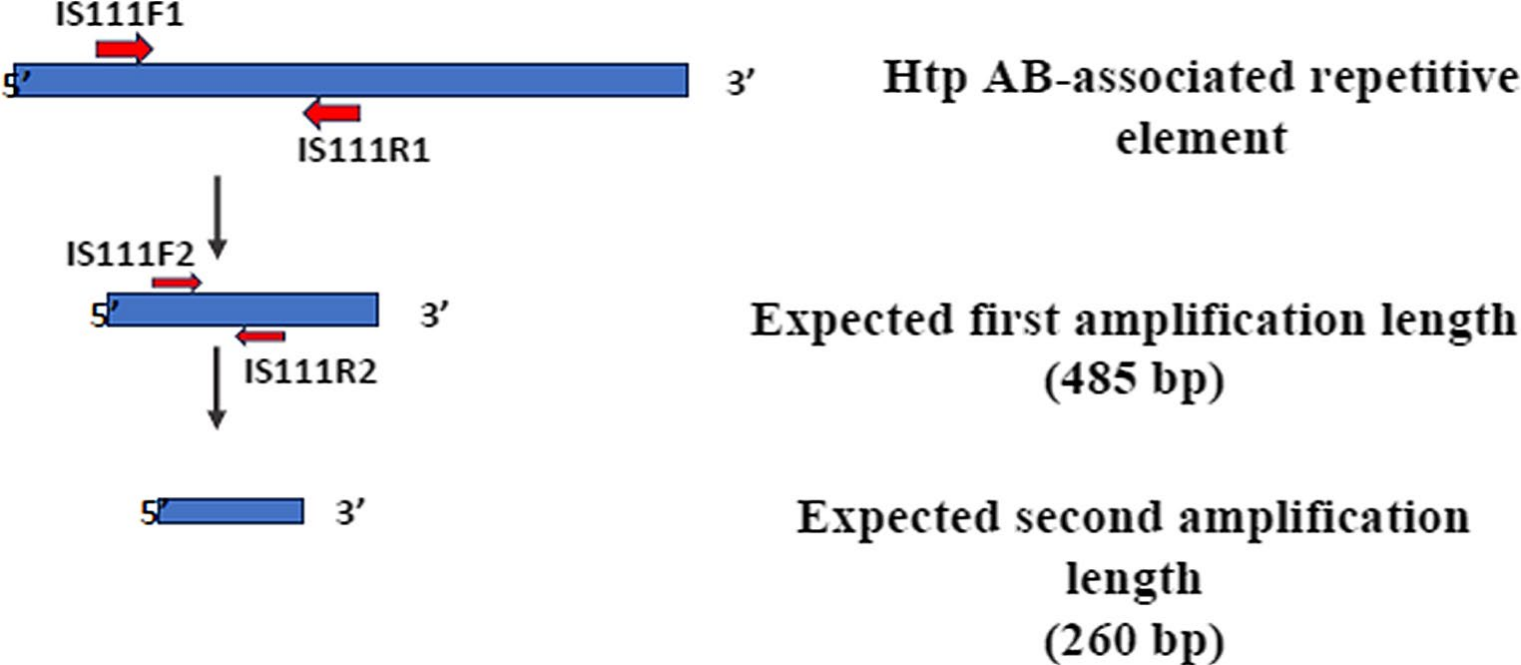
Diagrammatic representation of the 2 expected amplified regions of the Htp AB-associated repetitive element in *Coxiella burnetii* showing the 4 forward and reverse primer positions used (Abdel-Moein and Hamza 2017)

*Coxiella burnetii* detection was carried out by nested PCR sets of primers that target the htpAB-associated repetitive element (Abdel-Moein and Hamza 2017) (Fig. 3). The first amplification cycle was run using the primers IS111 F1 and IS111 R1, which amplify a 485-bp fragment, while the second (nested) PCR was performed with the IS111 F2 and IS111 R2 primers to amplify a 260 bp fragment (Abdel-Moein and Hamza 2017). A negative control containing PCR-grade water in the PCR master mix instead of DNA was included in every run to control possible contamination.

All primers used are tabulated in the Supplementary File (see supplement).

Ten microlitres of the amplification products were analyzed on a Novel Green Plus (20,000X concentration in DMSO; GeneDireX, Inc., USA) and stained on a 1.5% agarose gel in TAE buffer (0.04 M Tris-acetate, 0.002 M EDTA, pH 8) after horizontal electrophoresis at 90 V for 40 min (Zore et al. 1999; Santino et al. 2008). The produced bands were subsequently matched against 100 bp DNA Ladder H3 RTU (ready-to-use) (GeneDireX, Inc., US.)

## Results

### Infestation of ticks in the inspected animals

**Fig. 4:**
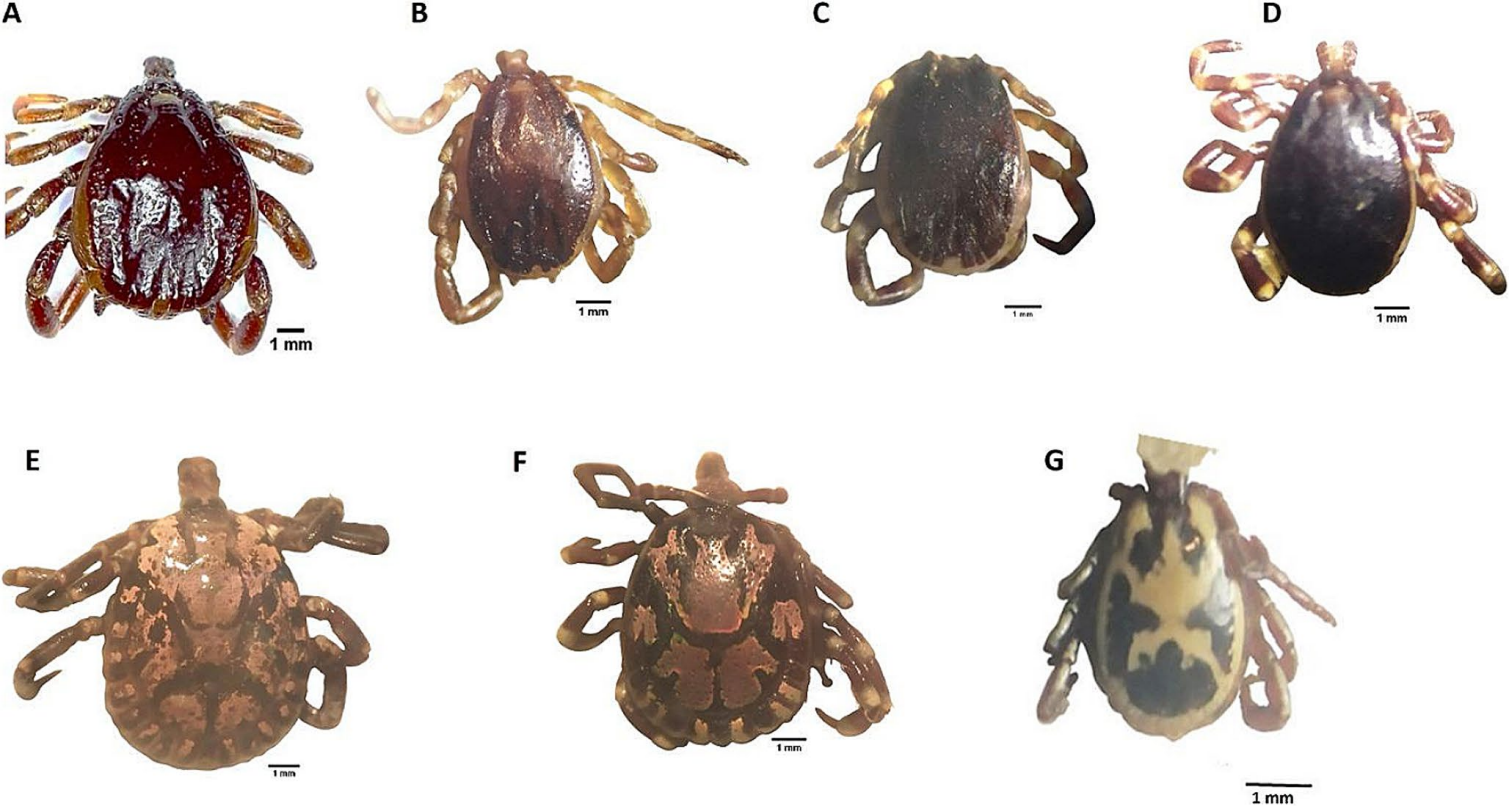
Dorsal view of the collected tick species **A** *Hyalomma dromedarii*; **B** *Hyalomma excavatum*; **C** *Hyalomma impeltatum*; **D** *Hyalomma rufipes*; **E** *Amblyomma marmoreum*; **F** *Amblyomma gemma*; **G** *Rhipicephalus pulchellus*

From 100 inspected camels, 1000 ticks were collected. Morphological identification of the collected ticks revealed seven ixodid species belonging to three genera: *Hyalomma*, *Amblyomma*, and *Rhipicephalus *(Table 1; Fig. 4). The genus *Hyalomma* was represented by four species; *Hyalomma dromedarii* was the most prevalent species and constituted 55.4% of the collected ticks, followed by *H. excavatum* (22%), *H. impeltatum* (11.6%) and *H. rufipes* (2.8%). The genus *Amblyomma* was represented by two species, *A. gemma* (2.8%) and *A. marmoreum* (2.7%), while the genus *Rhipicephalus* was represented by only one species, *R. pulchellus* (2.7%).

**Table 1:** Total number of tick species collected from inspected camels

Tick species	No. (%) of collected ticks
*Hyalomma dromedarii*	554/1000 (55.4%)
*Hyalomma excavatum*	220/1000 (22%)
*Hyalomma impeltatum*	116/1000 (11.6%)
*Hyalomma rufipes*	28/1000 (2.8%)
*Amblyomma gemma*	28/1000 (2.8%)
*Amblyomma marmoreum*	27/1000 (2.7%)
*Rhipicephalus pulchellus*	27/1000 (2.7%)

### Prevalence of detected tick-borne pathogens in blood from dromedary camels and brown rats

**Fig. 5:**
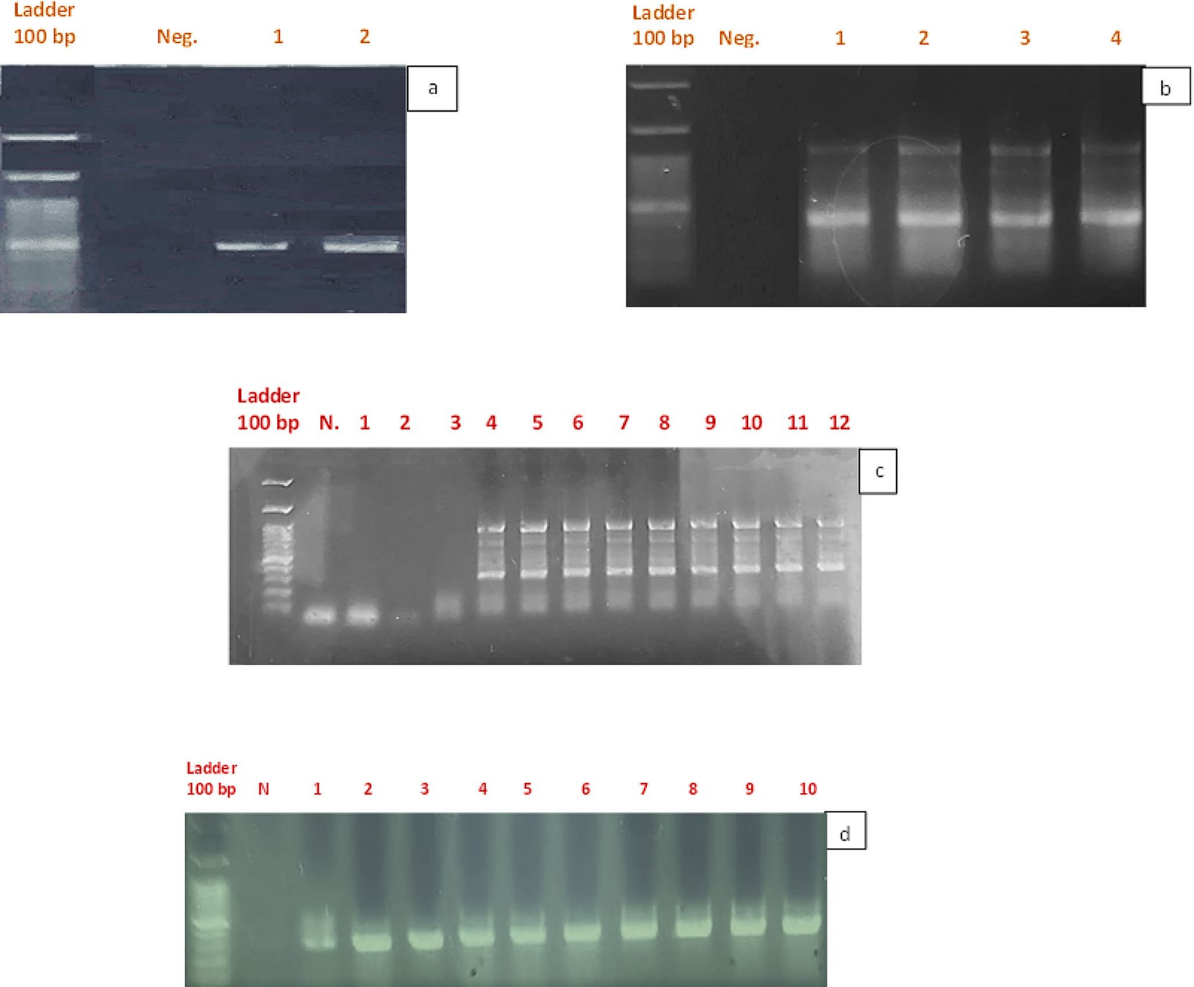
Molecular detection of TBP in ticks and blood from camels and brown rats. **a** *Borrelia burgdorferi;* Neg lane: negative control. Lane 1: camel blood sample. **b** *Borrelia miyamotoi;* Neg lane: negative control; Lane 1: blood of Brown rats; Lane 1: blood of camel; Lane 3: *H. impeltatum ticks; ***c** *Babesia* sp., N lane: negative control; lanes 1–3: negative PCR product of *Babesia* sp.; lanes 4–11: positive camel blood samples of *Babesia* sp., **d** *Coxiella burnetiid*, N: negative control; lanes 1–9: positive tick samples

Blood samples obtained from dromedary camels were positive for *Borrelia burgdorferi* (*n* = 66, 66%), *Borrelia miyamotoi* (*n* = 55, 55%), and *Babesia sp.* (*n* = 11, 11%) (Table 2; Fig. 5). However, all the samples were negative for *C. burnetii*. and coinfection with *Borrelia miyamotoi* and *Borrelia burgdorferi* was detected in 55 camels. These camels showed mixed infection with previous pathogens other than babesia in 11 camels out of 55 coinfected camels.

**Table 2:** Prevalence rate of tick-borne pathogens in dromedary camels and collected rodents based on molecular investigations

	Dromedary camels’ blood	Rodents’ blood
*Borrelia miyamotoi*	55%*	6%
*Borrelia burgdorferi*	66%*	0%
*Coxiella burnetii*	0%	0%
*Babesia* sp.	11.6%*	0%

*Coinfection detected in camel blood

Additionally, dromedary camels’ blood showed that 55 camels have a mixed infection with both *Borrelia sp.* and only 11 from 55 have coinfections with *Babesia* in addition to the above *Borrelia sp*.

Concerning blood, which was collected from rodents, only 6 out of 100 collected rodents were positive for *Borrelia miyamotoi* (*n* = 6, 6%) (Table 2; Fig. 5). PCR failed to detect DNA from other pathogens in the blood of the rodents.

### PCR-based infection rates of tick-borne microbes in collected ticks

Ticks were positive for only pathogens of borreliosis (*Borrelia miyamotoi* and *Borrelia burgdorferi)* and *Coxiella burnetii*. For borreliosis detection, *B. miyamotoi* was the primary species detected in *H. impeltatum* (*n* = 16, 12.5%), and *H. dromedarii* harbored *Borrelia burgdorferi* (*n* = 9, 1.6%). Interestingly, all the *H. dromedarii* ticks were infected with *Coxiella burneti*i (*n* = 554, 100%) (Table 3).

**Table 3:** PCR-Based infection rates of tick-borne pathogens in Collected ticks

Tick Species	Number of Ticks collected	Percentage of Positive Samples (Total Infection Rate)			
		*Borrelia miyamotoi*	*Borrelia burgdorferi*	*Coxiella burnetii*	*Babesia sp.*
*Rhipicephalus pulchellus*	27	0	0	(27) 100	0
*Am. gemma*	28	0	0	(9) 32.1	0
*Am. marmoreum*	27	0	0	(27) 100	0
*Hyalomma rufipes*	28	0	0	(17) 60.6	0
*H. excavatum*	220	0	0	(110) 50	0
*H. impeltatum*	116	12.5	0	(93) 80	0
*H. dromedarii*	554	0	1.6	(554) 100	0

**Fig. 6:**
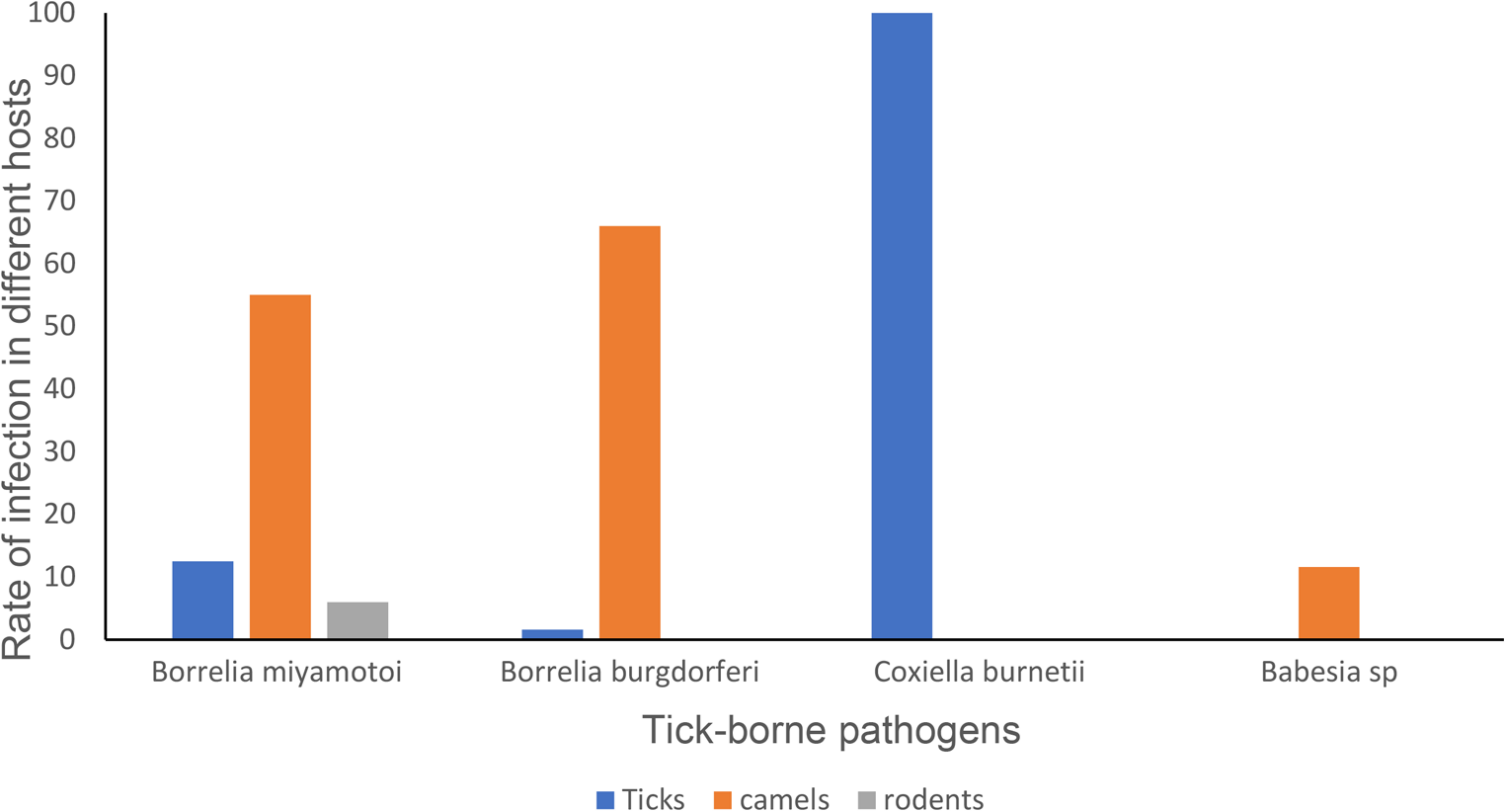
Rate of TBP infection among ticks, camels, and brown rodents

The information provided demonstrated the transmission cycle that occurs between camels, brown rats, and ticks and could be systematically observed in the case of *Borrelia* parasites (Fig. 6). However, in *Babesia* and *Coxiella*, it could be said that it is nonsystematic that may either interrupt the feeding of ticks or that some of the juvenile stages (larva and nymph) of these ticks acquire infection and not pass it to adults.

## Discussion

This tick-borne disease survey targeted the detection of potential vector-borne pathogens present in camels and accompanying ticks as well as in wild rats in contact with camels. Several factors contribute to the increase in tick-borne pathogens, including the geographic spread of ticks due to extensive livestock husbandry, the importation of animals from other regions, and the wide variety of wildlife species that sustain tick life cycles (Perveen et al. 2021). Pathogens and associated diseases are highly important to human and animal health (Daszak et al. 2000; Cunningham et al. 2017), and microbial and parasitic infections are ubiquitous in animal and human populations (Cunningham et al. 2017; Cable et al. 2017). The density of hosts, vectors and pathogens in a geographic area are key determinants of disease transmission (Martinez and Merino 2011).

Tick infestations in camels have a high economic cost because ticks considerably impact their health and productivity (Nazifi et al. 2011). In this study, 100 camels with ixodid tick infestations were examined, and 7 species of ticks from 3 genera were found to be collected from dromedary camels. There was one species in the *Rhipicephalus* genus, two in the *Amblyomma* genus, and four in the *Hyalomma* genus. Notably, the genus *Hyalomma* exhibited the highest prevalence, constituting 91.8% of the observed ticks. According to other studies (Okely et al. 2021; Abdel-Shafy et al. 2021; Barghash et al. 2016; Hassan et al. 2017), H. *dromedarii* is the most prevalent tick species on Egyptian camels, whether it is farmed or imported. In Egypt, *Amblyomma* species, *H. rufipes*, and *R. pulcullus* are nonendemic species, and their occurrence is mainly due to imported dromedary camels from Sudan, Ethiopia, Nigeria, and Somalia (El Kammah et al. 2001). Similar to the results of a study survey that covered 7 regions in Egypt with diverse climatic and social levels, the results revealed that Okely et al. (2021) detected almost the same species, with little difference in their frequency. *Amblyomma gemma*, 1%; *Hyalomma impeltatum*, 8%; *Hyalomma dromedarii*, 77%; *Hyalomma excavatum*, 10%; *Hyalomma rufipes*, 1.5%; *and Rhipicephalus pulchellus*, 1%. Okely et al. (2021) reported that dromedary camels had both the highest infestation rate and density of infestations (no. of collected ticks/no. of infested animals) 70% and 3.9, respectively. The shift in tick distribution may be attributed to increasing temperature, commonly described as global warming (Daniel et al. 2003; Jaenson et al. 2012; Medlock et al. 2013). An increase in climate temperature is thought to encourage the establishment of a constant outdoor population of Mediterranean tick species such as *R. sanguineus* (Duscher et al. 2015).

Along the western border of Egypt, viz. Libya, Abdulsalam et al. (2022) recognized the presence of *Hyalomma dromedarii* (83.12%) in camels only and *Hyalomma excavatum* (6.63%) in other animal species. In contrast, in southern Sudan, where it is considered the main gateway for the camel trade with Egypt and in contrast to the OIE rules, camels enter Egypt without sufficient quarantine, which provides a reasonable explanation for the presence of *Amblyomma marmoreum* and *Rhipicephalus pulchellus*, although they are nonendemic in Egypt. A long walking excursion is considered sufficient to rule out infection in animals (Elbayoumy et al. 2013). In a cross-sectional study (Shuaib et al. 2020) in which ticks were detected in three states in Sudan, 14 tick species belonging to *Amblyomma*, *Hyalomma*, and *Rhipicephalus* were the most common ticks. Additionally, among all the animal species, camels (16.9%) were the second most common after cattle in terms of tick burden. In terms of tick distribution, *H. rufipes* ranged from 0.2 to 22.4%. Other species, such as *H. impeltatum*, H. *dromedarii and Rhipicephalus sp*., were detected with low incidences ranging from 0.2% to approximately 36%. On the public level, Saudi Arabia is considered a shuttle pathway against Egypt. The tick distribution and abundance data from Riyadh agreed with our results, where two genera were identified, *Hyalomma* 68.3% and *Rhipicephalus* 31.7%, with a predominance of *H. dromedarii* (39.9%) (Alanazi et al. 2019). Studies on ticks and associated pathogens in camels collected from different regions and countries have reported that *H. dromedarii* is the main species parasitizing dromedary camels in addition to other species with minor incidences. In Riyadh Province, Saudi Arabia (Alanazi et al. 2020), H. *dromedarii* was approximately 76.4%, followed by *H. impeltatum* (23.3%) and *H. excavatum* (0.3%). However, in the UAE, Perveen et al. (2021) reported that almost 100% of ticks collected from camels were *H. dromedarii*. Hussain et al. (2023) recorded eleven tick species belonging to 4 genera, with overall 14.3% infestation in eight animal species and 19.6% in camels. This study was in accordance with our results, where *Hyalomma* spp. were the predominant species, with an average of 47.4 − 70%. However, they recorded *Rhipicephalus* spp. with a high incidence, reaching 69.7%, infesting animal species other than camels. In a study on tick distribution in Kazakhstan (Sang et al. 2021), they noted species of *Hyalomma* on camels other than those recorded in this study, which ranged from 9.95 to 19.36%.

Due to the expanded geographical range of tick populations worldwide, the prevalence and transmission of TBDs are increasing. Although *B. burgdorferi* is transmitted among several host species, infected hosts differ considerably in their capacity to infect feeding ticks (Jaenson and Tälleklint 1992; Brunner et al. 2008; Gandy et al. 2022). In the present study, *B. burgdorferi* was detected in camels (66%, 66/100), and *B. burgdorferi* was detected in only 9 samples of ticks (1.6%, 9/554). Jaenson and Tälleklint (1992) reported that roe deer serve as a principal blood source for *Ixodes ricinus* but were unable to infect feeding ticks with *B. burgdorferi*. The authors proposed that deer may possess a mechanism that prevents them from being infected by ticks. Few studies have detected *B. burgdorferi* in camel blood (Ben Said et al. 2016; Ashour et al. 2023), and the potential role of camels in pathogen transmission and prediction of disease risk is still unknown. *B. miyamotoi* was isolated from *Ixodes persulcatus*, and the first case of *B. miyamotoi* infection in a human was discovered in Russia in 2011 (Platonov et al. 2011). Nevertheless, there have been no reports of human *B. miyamotoi* infections in Egypt. Most recently, in Egypt, *B. miyamotoi* was first identified in 6.8% of camel blood samples using the glpQ gene (Ashour et al. 2023). These results support our finding that 6% of rodent blood was PCR positive for *Borrelia miyamotoi*. However, DNA from other targeted pathogens has not been detected in rodents. These results are in accordance with those of (Khanakah et al. 2006; Schmidt et al. 2014), who detected *Borrelia*-specific DNA in two local studies on rodents, which ranged from 14.8 to 53.3% in Eastern Dumas et al. (2022) detected both *B. burgdorferi* and *B. miyamotoi* in biopsies from mice. A high prevalence of *Borrelia burgdorferi* has been reported in synanthropic murid rodents (Solís-Hernández et al. 2016). The overall infection rate in rodents was 36.5%. *Rattus rattus* had 17.2% infection with the spirochete *Borrelia burgdorferi* s.l. Olivieri et al. (2021) detected *Borrelia* in only one tick (*H. rufipes*) and the pathogenic *Coxiella burnetii* in one specimen of *Rh. pulchellus.*

The absence of *Babesia* and *Theileria* species DNA in the collected ticks is consistent with the low prevalence of these pathogens reported in previous studies carried out on tick-borne diseases of camels (Al-Deeb et al. 2015; Alanazi et al. 2020). In the present work, the absence of positive ticks for the examined pathogens indicates a low risk of exposure via tick bites. Further studies are required to obtain a thorough epidemiological picture of the area, including more farms for an expanded survey of the camel population.

Q-fever has received limited attention due to the perceived low occurrence of this disease in both humans and animals. Nevertheless, one of the major challenges lies in its asymptomatic nature. (Tissot-DuPont and Raoult 2008). *H. dromedarii* could also be considered a potential vector for maintaining the endemism of *C. burnetii* in nature but is not essential for its natural cycle or endemism maintenance in livestock. (Gargili et al. 2017; Abdullah et al. 2018). This survey supports previous results in which *C. burnetii* DNA was detected in all the collected ticks (Abdullah et al. 2018; Alanazi et al. 2020), however, Q-fever pathogen DNA was not detected in the blood of camels or brown rats. In contrast, El Tigani-Asil et al. (2021) detected the htpB gene of *C. burnetii* (3.2%) and the 18 S rRNA gene of *Babesia* sp. (2.1%) in camel blood in the UAE.

The reservoir status of various vertebrate species was clarified by Gern and coauthors, who, although focused upon *Borrelia* transmission alone, promoted the value of xenodiagnoses as the gold standard for determining the ability to serve as a reservoir host (Gern et al. 1998). Successful co-feeding transmission can occur when noninfected ticks cluster into preferred host feeding sites, such as around the ears of mammals or the bill area of birds, in the absence of detectable systemic infection. This clustering is further enhanced by the production of aggregation pheromones by some ticks (Randolph et al. 1996). Collectively, these approaches provide us with tools to evaluate the ability of vertebrates to influence the coinfection of ticks. Defining reservoir host competency based upon evidence of systemic infection has been increasingly challenging. Many species deemed refractory to pathogens may indeed contribute to the ecology of tickborne pathogens through tick co-feeding as opposed to systemic routes (Belli et al. 2017; Ogden 2017; Randolph et al. 1996; States et al. 2017).

Most rodents are trapped in peridomestic environments because of the plentiful food supplies available in their surroundings. This suggests a high chance of parasite exchange among the epidemiologic triad, pathogens, rodent–human communities, and wildlife. Rodents can transmit various pathogens belonging to the genus Apicomplexan and spirochetes during grooming by ingesting ticks as prey or via fecal drops to be ingested by the vertebrate host (Murata et al. 1993; Baneth et al. 2003). The fluctuation of rodent populations is considered to occur seasonally when rodents have a yearly cycle. In addition to its biogeography, we anticipate that rodent self-grooming and collection methods affect the detection of ticks and their stages in rodents. The role of rodents in the transmission of vector-borne pathogens has not been determined.

A study revealed discrepancies in the associations between rodents and tick abundance and tick life stages and between tick abundance and the abundance of vector-borne pathogens, but these findings are controversial (Krawczyk et al. 2020). They hypothesized that additional factors, such as the synchrony of activity and infection, play a role in pathogen dynamics in rodents and ticks. Nevertheless, a positive density dependence for all rodent- and tick-associated tick-borne pathogens was found (Aminikhah et al. 2021). Additionally, tick–host–pathogen interrelationships are changing due to the shifting population density of ticks, tick-borne infections, and changing density of hosts (de la Fuente et al. 2016; Hoogstraal and Valdez 1980; Wikel 2018). Understanding the epidemiology of tick-borne diseases, especially their transmission dynamics through tick vectors, is essential for the formulation of efficient control strategies (Wikel 2018; Morzaria et al. 1992).

Regarding coinfection, as the actual tick that transmits infection is rarely available, we argue that evidence of a tick bite serves only to demonstrate exposure to tick-infested habitats and that coinfection might follow the bite of one or more ticks either simultaneously or following sequential transmission events (Cutler et al. 2021). From a clinical perspective, we define tick-borne coinfections as those acquired by transmission from one or multiple ticks either following a single exposure or multiple sequential exposures. Coinfections present diagnostic challenges, and pathogens might behave synergistically, indifferently, or antagonistically within their respective hosts, thus modulating disease severity (Belongia 2002; Diuk-Wasser et al. 2016; States et al. 2017).

## Conclusion

This study sheds light on the cycle of tick-borne pathogens among tick species that parasitize camels and rodents as reservoir hosts. Camels, either farmed or imported, harbor several neglected, emerging, and re-emerging TBPs. Additionally, *Borrelia miyamotoi* was detected for the first time in three hosts: camels, ticks, and rodents. This is significant because if any of the ticks are one-host life cycle species, it would not be surprising that the brown rat does not play a role in zoonotic pathogen transmission. However, further research is needed to determine the dynamics of the rodent host population that drive zoonotic infections caused by tick-borne pathogens.

## Electronic supplementary material

Below is the link to the electronic supplementary material.

Supplementary Material 1

## Data Availability

No datasets were generated or analysed during the current study.
