# Supplementary material for: Molecular detection of some zoonotic tick-borne pathogens in ticks collected from camels (Camelus dromedarius) as hosts and wild rodents as potential reservoirs
Source: Vet Res Commun. 2024 Aug 15;48(5):3197–207. doi: 10.1007/s11259-024-10488-9 (PMC11442481; doi:10.1007/s11259-024-10488-9)
Supplement: Supplementary file 1 — Supplementary Material 1 [file 11259_2024_10488_MOESM1_ESM.docx]

**Table 1. Primers sets of detected tick-borne pathogens**

| **Pathogen** | **Target gene** | **Primer Sequences 5’-3'** | **PCR conditions** | **Expected amplicon size (bp)** | **Reference** |
| --- | --- | --- | --- | --- | --- |
| *Borrelia burgdorferi* | 16S rRNA gene | **BbF**  5′-GGGATGTAGCAATACATTC-3′  **BbR**  5′-ATATAGTTTCCAACATAGG-3 | - 94◦C for 1 min, followed by 35 cycles of 95◦C for 1 min  - 50◦C for 1 min  - 72◦C for 1.5 min  - then final extension at 72◦C for 7 min | 577 bp | Marconi & Garon, 1992 |
| *Borrelia miyamotoi* | 16S-23SDNA gene | **S1**  CGATAGGATCCAAGCATGCAAGTCAAACG  **S2**  CGATAGAATTCTAACTTCCCCTATCAGAC | - 95◦C for 5 min, followed by 35 cycles of 95◦C for 30 s  - 50◦C for 45 s  - 72◦C for 45 s,  - then final extension at 72◦C for 7 min. | 450 bp | Reiter et al., 2015 |
| *Coxiella burnetii* | Htp AB-associated repetitive element | **IS111 F1**  5-TACTGGGTGTTGATATTGC-3  **IS111 R1**  5-CCGTTTCATCCGCGGTG-3 | - 95◦C for 3 min, followed by 40 cycles of:  - 95◦C for 30 s, 52◦C for 30 s  - 72◦C for 1 min  - then final extension at 72◦C for 4 min | 485 bp | Abdel-Moein, & Hamza, 2017 |
|  |  | **IS111 F2**  5-GTAAAGTGATCTACACGA-3  **IS111 R2**  5-TTAACAGCGCTTGAACGT-3 | - 95◦C for 3 min, then 30 cycles of  - 95◦C for 30 s,  - 52◦C for 30 s  - 72◦C for 30s  - with final extension at 72◦C for 4 min. | 260 bp |  |
| Babesia sp. | Babesia ss-rDNA | **Babl**  5'-CTTAGTATAAGC'1'11ATACAGC-3'  **Bab4**  5'ATAGGTCAGAAACTTGAATGATACA-3' | - 95◦C for 1 min, then 35 cycles of  - 94◦C for 1m,  - 55◦C for 1m  - 72◦C for 2m  - with final extension step at 72◦C for 4 min. | 238 bp | Persing et al., 1992; Wei et al., 2001 |
